# Titin stiffness modifies the force-generating region of muscle sarcomeres

**DOI:** 10.1038/srep24492

**Published:** 2016-04-15

**Authors:** Yong Li, Patrick Lang, Wolfgang A. Linke

**Affiliations:** 1 Department of Cardiovascular Physiology, Ruhr University Bochum, Germany

## Abstract

The contractile units of striated muscle, the sarcomeres, comprise the thick (myosin) and thin (actin) filaments mediating active contraction and the titin filaments determining “passive” elasticity. We hypothesized that titin may be more active in muscle contraction by directly modulating thick-filament properties. We used single-myofibril mechanical measurements and atomic force microscopy of individual sarcomeres to quantify the effects of sarcomere strain and titin spring length on both the inter-filament lattice spacing and the lateral stiffness of the actin-myosin overlap zone (A-band). We found that strain reduced the lattice spacing similarly in sarcomeres with stiff (rabbit psoas) or compliant titin (rabbit diaphragm), but increased A-band lateral stiffness much more in psoas than in diaphragm. The strain-induced alterations in A-band stiffness that occur independently of lattice spacing effects may be due to titin stiffness-sensing by A-band proteins. This mechanosensitivity could play a role in the physiologically important phenomenon of length-dependent activation of striated muscle.

Force generation in muscle cells depends on the interaction between actin and myosin as the main constituents of the thin and thick myofilaments in the contractile units, the sarcomeres. The myofilaments are linked by the giant protein titin, which determines the “passive” elastic properties of the myocyte. The sarcomere’s contractile force, at a given concentration of activator Ca^2+^, is affected by the stretch state: at higher sarcomere length (SL) the force is greater than at lower SL, at least when a near-optimal overlap of the thin and thick filaments is maintained. This length-dependent activation (LDA) underlies, in part, the physiologically important autoregulation of the heart known as the Frank-Starling law, which describes the increase in contractility with increased filling^1^,^2^. Conversely, a reduction in SL is associated with shortening deactivation. In the beating heart these sarcomere dynamics are important in the modulation of cardiac contractility^3^. The SL-dependent regulation of contractile force is not unique to cardiac muscle but is also seen, to a lesser degree, in skeletal muscle^4^.

While it is well known that sarcomere stretching immediately increases the Ca^2+^ sensitivity of the contractile apparatus, the molecular mechanisms behind the LDA phenomenon are incompletely understood. A determinant could be the lateral spacing between the thin and thick filaments^5^,^6^,^7^. Stretch of the sarcomere reduces this spacing (Fig. 1a), which may facilitate actin-myosin interaction and force generation. The factors that stabilize the filament lattice at rest and shrink it laterally on stretch were proposed to include the electrostatic forces between the charged actin and myosin surfaces, the radial stability of the M-band and Z-disk regions of the sarcomere, and the stiffness of titin^8^. However, this explanation for the mechanism of LDA has been challenged by the demonstration that myofilament Ca^2+^ sensitivity depended only on SL and not on inter-filament spacing *per se*^1^,^9^.

Among the proteins suggested to contribute to LDA, titin seems to be a particularly strong candidate^2^,^10^,^11^,^12^,^13^,^14^. The stiffness of titin could modulate LDA by adding to lattice compression via a radial force component (*F*_R_) that arises out of stretching the titin springs in the sarcomere (Fig. 1a, inset). This component appears because the titin springs bind to the actin filament at the Z-disk and to myosin at the edge of the A-band, such that they are not fully aligned with the myofibril axis (Fig. 1a). Alternatively, the elastic force developed on stretching titin could directly modify the structural arrangement or conformation of proteins in the A-band region, where actin and myosin interact. The aim of this study was to test at the level of the individual sarcomere whether the stiffness of titin alters A-band mechanical properties and if so, whether or not this effect is explainable by titin-dependent alterations in myofilament lattice spacing. Our results suggest that titin could mediate LDA through strain-induced stress sensing at the actin-myosin overlap zone.

## Results and Discussion

### Titin isoform size and passive stress in uncompressed vs. dextran-compressed myofibres

**Figure 1:**
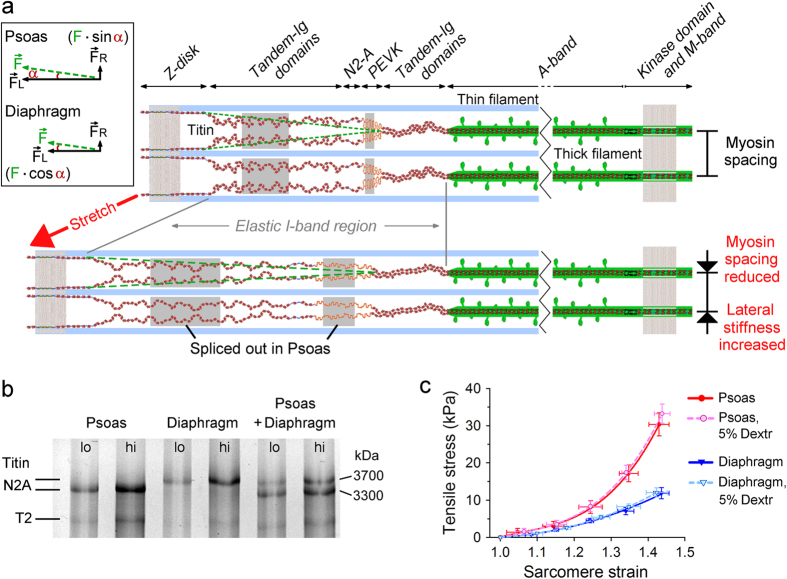
Titin size differences cause different mechanical properties of muscle sarcomeres. (**a**) Three-filament model of a half-sarcomere highlighting titin. Strain extends the titin springs, which are shorter in psoas than in diaphragm muscle. (Inset) Titin-based force components in longitudinal (*F*_*L*_) and radial direction (*F*_*R*_) arising from sarcomere strain, due to the slightly oblique positioning of I-band titin (broken green lines). (**b**) Titin gel showing different molecular size in psoas and diaphragm. lo, low loading; hi, high loading. N2A, full-length titin; T2, degraded/small titin. (**c**) Stress-strain relationships of single skinned myofibres in relaxing buffer, in presence or absence of 5% dextran. Strain was calculated from SL measured by laser diffraction; strain of 1.0 is slack; strain of 1.45 is 3.0–3.2 μm SL. mean ± SEM (n = 4 fibres per condition), fits are polynomial regressions.

Two different rabbit muscle types were used for the experiments, one expressing a relatively small titin isoform (psoas; M_W_ ~ 3.3 MDa), the other a large titin isoform (diaphragm; M_W_ ~ 3.7 MDa) (Fig. 1b). The size difference arises due to alternative splicing of modules in the titin spring segment (Fig. 1a)^15^. By performing force and sarcomere length (SL) measurements of skinned myofibres in relaxing buffer we found a 2–3 times higher passive tension in psoas vs. diaphragm sarcomeres (Fig. 1c). However, the myofilament lattice becomes expanded with skinning, compared to the *in vivo* situation, which could affect titin-based stiffness. Therefore, we also measured the passive stress-strain relationships in the presence of an osmotic compressing agent, dextran T-500, which at high concentrations (15%) was shown to modestly increase the passive tension of skinned cardiac muscle strips^16^. We supplemented the relaxing buffer with 5% dextran (lattice compression, 4.7 kPa), because myofilament calcium sensitivity is stable within the 2–6% concentration range^17^ and because a dextran concentration of 5% has frequently been used by others to normalize the lattice spacing of skinned skeletal myofibres and to mimic the physiological situation^18^,^19^,^20^. We found that 5% dextran did not significantly alter the passive stress-strain curve of either fibre type (Fig. 1c).

### Strain causes similar A-band compression in psoas and diaphragm sarcomeres

**Figure 2:**
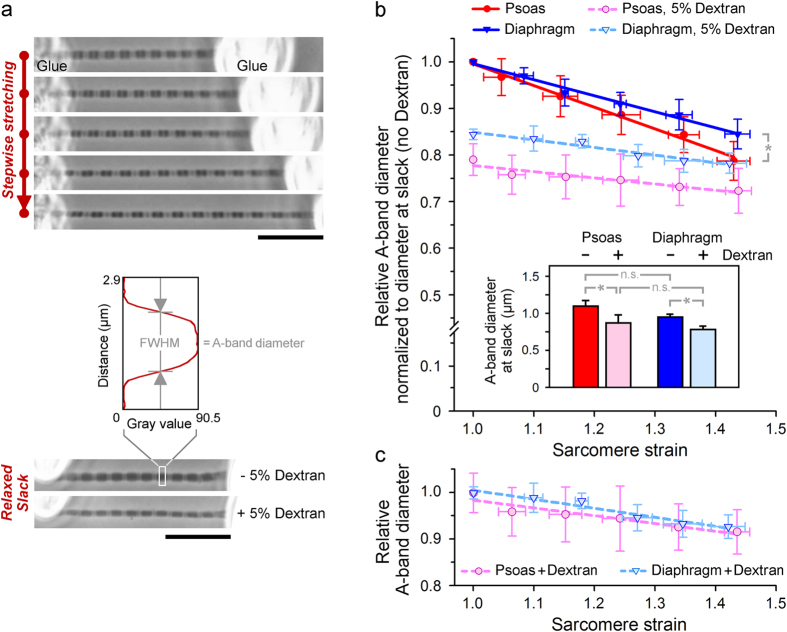
Strain-dependent decrease in A-band diameter of single myofibrils in relaxing buffer with or without 5% dextran. (**a**) Phase-contrast images of psoas myofibrils at different stretch states (top), in absence/presence of dextran at slack SL (bottom), and measurement of A-band diameter from the full-width at half-maximum (FWHM) on intensity profiles; scale bars, 10 μm. (**b**) Effect of strain on mean A-band diameter indexed to diameter at slack in absence of dextran. mean ± SEM (n = 6 myofibrils; 5 A-bands/myofibril); fits are linear regressions. *p < 0.05. (Inset) Mean A-band diameter of psoas and diaphragm myofibrils at slack SL in absence or presence of 5% dextran. (**c**) Effect of strain on mean A-band diameter of dextran-compressed myofibrils, expressed relative to diameter at slack in presence of dextran.

Strain is expected to decrease the width of a muscle fibre^8^. We wanted to know whether sarcomere strain also reduces the width of the A-band in the isolated myofibril, as predicted, e.g., from the presence of titin’s radial force component (Fig. 1a). Because fibre width and myosin lattice spacing are linearly related in skinned rabbit psoas fibres^19^, we assumed that the relationship between A-band diameter and lattice spacing is also linear. We measured the A-band diameter of non-activated single rabbit psoas or diaphragm myofibrils during stepwise stretching from slack SL (2.1–2.3 μm; strain, 1.0) up to ~145% slack SL (3.0–3.2 μm) (Fig. 2a), the latter of which is at the high end of the physiological SL range in rodent muscles^21^. For sarcomeres in relaxing buffer lacking dextran, the A-band diameter (mean value at slack SL, 1.10 μm for psoas and 0.95 μm for diaphragm, with no significant difference; Fig. 2b, inset) successively decreased with increasing strain, reaching ~79% and ~85% of the initial value at the highest strain level, in psoas and diaphragm, respectively (Fig. 2b). The mean values were well fit by linear regressions and agreed with those obtained by others for the effect of SL-increase on the width of skinned myofibres^5^,^20^,^22^. We observed a slightly larger decrease in psoas vs. diaphragm A-bands.

Upon addition of 5% dextran to the relaxing buffer in order to normalize the lattice, the A-bands became thinner by 15–20% at slack SL (Fig. 2a, bottom; Fig. 2b). This finding is consistent with previous measurements on permeabilized myofibres or myofibrils showing similarly reduced widths in response to osmotic compression by these low dextran concentrations^19^,^20^,^23^,^24^. In our hands, the dextran-effect was somewhat larger in psoas than in diaphragm sarcomeres. Stretch of the dextran-compressed myofibrils further reduced the A-band diameter. However, the negative slopes of the fit curves to the means were less steep than before osmotic compression (Fig. 2b). Importantly, the proportional decrease in A-band diameter at comparable strain levels was now identical in the two myofibril types (Fig. 2c). Elsewhere, skinned rat skeletal and cardiac muscle fibres, which differed greatly in titin-based stiffness, were studied by X-ray diffraction for stretch-dependent changes in the myosin lattice spacing^4^. The relative reduction in lattice spacing that was found with strain was the same in the different muscle types, while the absolute values differed. Another study reported a decrease in inter-filament lattice spacing with titin-based spring force, for skinned rabbit psoas fibres; however, the effect was much reduced following osmotic compression by dextran^11^. We conclude that the strain-induced reduction in A-band diameter or lattice spacing does not depend much on the stiffness of the titin spring, in particular so in sarcomeres in which the lattice is modestly compressed (as under physiological conditions).

### The lateral thick-to-thick filament spacing in individual A-bands deduced from AFM images

**Figure 3:**
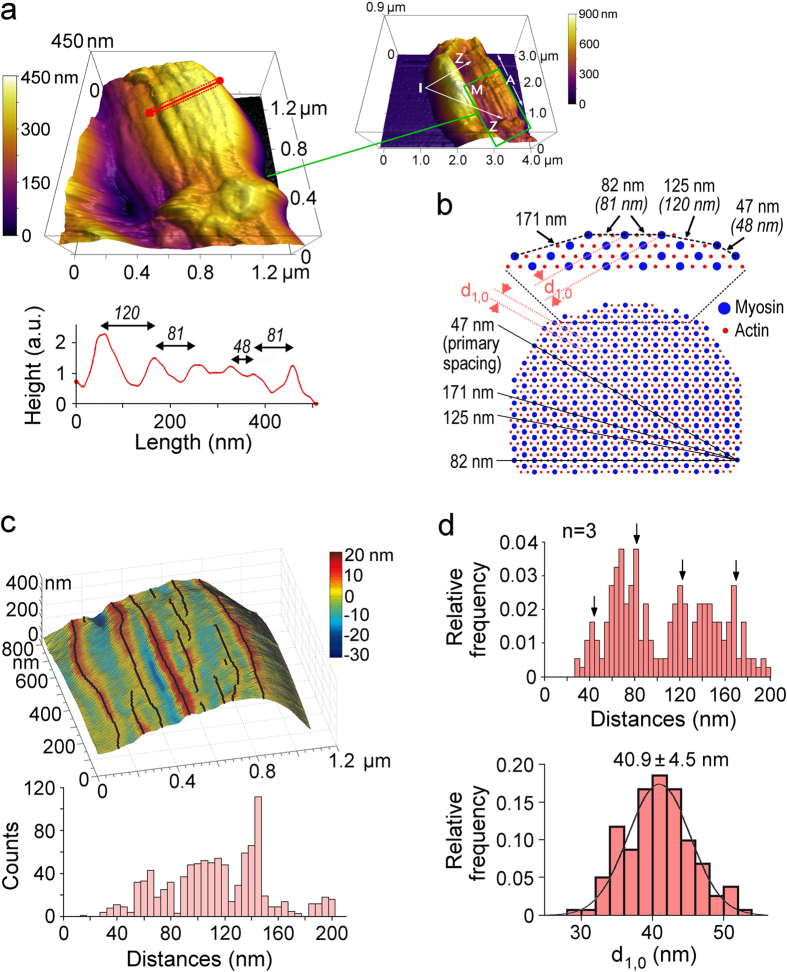
Detection of the myosin spacing in relaxed sarcomeres by AFM. (**a**) 3D height-image of psoas half-sarcomere in relaxing buffer at slack SL (tapping mode); pixel size, 5.5 nm. On the profile plot (bottom), irregularly spaced peaks likely represent myosin filaments. Measured peak-to-peak distances are indicated. (Inset) 4 × 3 μm overview image (pixel size, 19.5 nm) of the same myofibril; sarcomere bands (Z, I, A, M) are marked. (**b**) Scheme of a cross-sectioned actin-myosin lattice. Magnified detail shows values for the diverse thick-to-thick filament spacings expected from a 47 nm primary spacing (A_P_ = 2/√3 × d_1,0_, with d_1,0_ = 40.3 nm (from X-ray diffraction)). Distances measured by AFM are in parentheses. Black lines cutting through the lattice represent 4 planes intersecting along the periphery of the myofibril where the myosin spacing is least. (**c**) Identification of putative myosin filaments via peak-detection algorithm. Top: reconstruction of the AFM image (A-band) depicted in (**a**); detected peaks (100 line scans) are marked and appear as black lines. Bottom: histogram showing all nearest peak-to-peak distances on the image. (**d**) Top: histogram showing all nearest peak-to-peak distances of detected putative myosin filaments, measured on AFM height images of 3 different relaxed psoas A-bands at slack SL. Arrows point to thick-to-thick filament lattice spacings expected at the myofibrillar surface based on a 47-nm primary spacing. Bottom: histogram of the d_1,0_ distribution inferred from the data in the top panel. The line is a best Gaussian fit giving d_1,0_ = 40.9 ± 4.5 nm (mean ± SD).

Since the A-band diameter is only an indirect measure of inter-filament lattice spacing, we wanted to obtain information about the thick-to-thick filament spacing in our single myofibrils. The aim here was to compare the lattice spacing in the single myofibrils, which have been removed from the crowded cellular environment, to that in muscle cells/bundles as determined previously by X-ray diffraction. To this end, we employed the AFM following an approach developed for insect myofibrils^25^. We chose rabbit psoas for these measurements, since values of myosin lattice spacing are readily available for this muscle type from X-ray diffraction experiments. In AFM tapping mode we acquired high-resolution height images of non-stretched myofibrils firmly adhering to a cover glass and bathed in relaxing buffer (Fig. 3a). The sarcomeric Z-disk, I- and A-bands could readily be distinguished and filamentous structures running axially along the A-band were clearly resolved. The peak-to-peak lateral distances between these elevated structures were analyzed on profile plots perpendicular to the myofibril axis (Fig. 3a). In the example shown, the observed inter-filament distances were 120 nm, 81 nm, and 48 nm. These values agree well with those expected for the thick-to-thick-filament spacings measurable on the myofibrillar surface (Fig. 3b). The diversity in spacings is due to the intersection of different planes of the thick-to-thick filament lattice at the surface of the myofibril^25^. Calculation of the spacing along these planes is based on a 47-nm primary spacing, A_P_, calculated as A_P_ = 2/√3 · d_1,0_, where d_1,0_ is the interfilament distance (Fig. 3b). We used a d_1,0_ value of 40.3 nm, which is from X-ray diffraction experiments and applies to relaxed rabbit psoas muscle at ambient temperature and SL ~ 2.2 μm^26^. A d_1,0_ value of ~40 nm has consistently been found for non-stretched, skinned rabbit psoas fibres under comparable buffer conditions^19^,^23^,^27^,^28^. Taken together, it is very likely that the filamentous structures running axially along the A-bands on our AFM images are the myosin thick filaments.

For a more comprehensive analysis of the myosin pattern in our AFM images we applied an algorithm that detected the elevated longitudinal structures in each line scan and followed them in 50–100 line scans/image to cover half of an A-band (Fig. 3c, top). Using this algorithm, we measured all nearest inter-filament distances in a given half-A-band (Fig. 3c, bottom). From the data shown in the histogram we inferred the primary spacing A_P_ and a d_1,0_ value of 43.2 ± 7.2 nm (mean ± SD). We applied this approach to 3 high-quality images of different psoas A-bands at 2.2–2.3 μm SL in relaxing buffer and pooled the values in a histogram (Fig. 3d, top). We found distinct peaks, among others, for inter-filament distances of approximately 47 nm (the expected primary spacing), 82 nm, 125 nm and 171 nm. From these data we generated a binned histogram for the distribution of calculated d_1,0_ values, which showed a mean d_1,0_ of 40.9 ± 4.5 nm (Fig. 3d, bottom). Thus, the myosin spacing in our single isolated psoas myofibrils is indistinguishable, within experimental error, from that reported in X-ray diffraction studies for relaxed (skinned, non-stretched) psoas muscle cells. These results suggest that the normal inter-filament lattice spacing is maintained in single isolated myofibrils dissected out of their cellular environment.

### Lateral stiffness maps of sarcomeres by AFM force mapping

**Figure 4:**
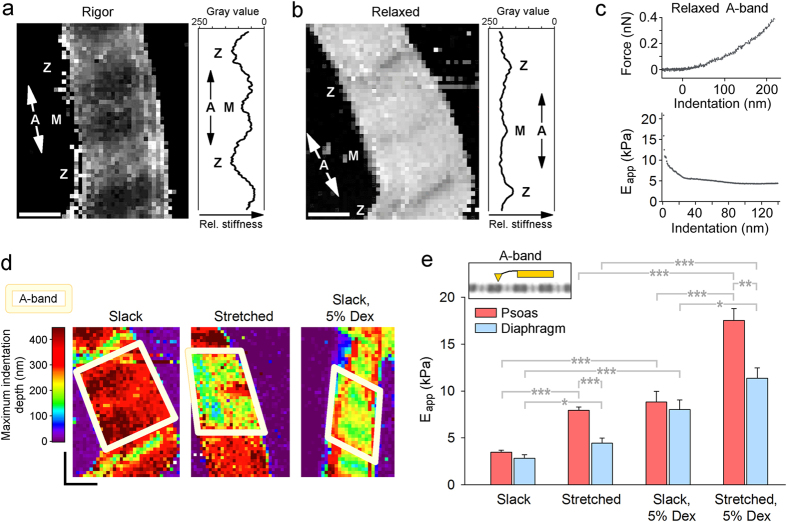
AFM-based force mapping of sarcomeres reveals differences in lateral stiffness. (**a**,**b**) Stiffness maps of non-stretched psoas sarcomeres at maximal indentation under a trigger force of 400 pN; (**a**) in rigor; (**b**) in relaxing buffer. Pixel size, ~80 nm; scale bars, 0.8 μm. Plots on right show stiffness profiles in longitudinal direction averaged over 10 pixels (darkest regions indicate smallest indentations or stiffest areas). Sarcomere bands (Z, A, M) are indicated. (**c**) Representative force-indentation curve (top) and pointwise apparent modulus (E_app_) vs. indentation depth relationship (bottom) for relaxed psoas A-band. (**d**) Stiffness maps of psoas sarcomeres at slack SL in relaxing buffer (left), after stretch in relaxing buffer (middle), and after dextran-treatment at slack (right). The A-band region is highlighted. The color scheme indicates maximum indentation depths at a given trigger force; scale bars, 0.8 μm. (**e**) Pointwise apparent modulus (at 60–80 nm indentation depth) of psoas and diaphragm A-bands in relaxing buffer at slack SL (n = 12 − 13 sarcomeres); ~145% stretch (n = 11 sarcomeres); slack SL, 5% dextran (n = 7 sarcomeres); and ~145% stretch, 5% dextran (n = 6 − 7 sarcomeres). mean ± SEM; *p < 0.05; **p < 0.01; ***p < 0.001; ANOVA/Bonferroni-adjusted t-test.

We reasoned that increasing the stiffness of titin in sarcomeres could affect the A-band region, (i) because the reduction in lattice spacing due to titin’s radial force component promotes the repulsive forces between the charged actin and myosin filament surfaces, (ii) and/or because the strained titin directly affects the conformation or structural arrangement of proteins in the overlap zone of thick and thin filaments. We speculated that these putative titin stiffness-dependent alterations could increase the lateral stiffness of the A-band. To measure lateral stiffness we used AFM force mapping, which monitors cantilever deflection in response to sample indentation^25^,^29^,^30^,^31^. Force maps consisting of 2,500 (50 × 50) force-indentation curves/image were generated on individual sarcomeres bathed in relaxing buffer, and occasionally also in “rigor” buffer lacking ATP (to maximize actin-myosin interactions). Representative force maps of psoas sarcomeres demonstrated much higher lateral stiffness under rigor compared to relaxing conditions (Fig. 4a,b), consistent with earlier findings^32^. We could distinguish regions of different stiffness along the sarcomere. In rigor, the A-band was the stiffest, followed by the M-band and the Z-disk (Fig. 4a). In relaxing buffer, the sarcomere was generally softer than in rigor and the Z-disk was the stiffest, followed by the M- and A-bands (Fig. 4b). For a convenient quantification of lateral stiffness at a given indentation we calculated the pointwise apparent modulus, E_app_^33^, from the force-indentation curves (Fig. 4c). Under relaxing conditions the E_app_ for A-bands decreased with indentation and reached a quasi-plateau beginning at 60–80 nm indentation depth; only the value at the plateau was used for further analysis. We prepared single myofibrils expressing stiff (psoas) or compliant titin (diaphragm) and measured E_app_ both at slack SL and following a ~45% stretch (SL, 3.1–3.2 μm), with or without 5% dextran. The stretch was accomplished under a phase-contrast microscope, the ends of the stretched myofibril were glued to a cover glass, and the sample (in relaxing buffer) on the cover glass was transferred to the AFM for force mapping (Fig. S1a). Force maps of stretched sarcomeres in the absence of dextran, as well as those of dextran-treated sarcomeres at slack SL, showed lower indentability reflecting higher A-band lateral stiffness compared to slack sarcomeres in the absence of dextran (Fig. 4d).

### Titin stiffness is an important determinant of A-band lateral stiffness

In the absence of dextran, the mean E_app_ of relaxed psoas and diaphragm A-bands at slack SL was identical, ~3 kPa (Fig. 4e). Addition of 5% dextran increased E_app_ by a factor of ~3 in both myofibril types. While an increase in myofibril lateral stiffness with dextran treatment was reported before^32^, our results show that modest compression of the myofilament lattice increases A-band lateral stiffness independently of titin spring length, at least in unstretched sarcomeres. In contrast, a 45% stretch affected E_app_ of psoas and diaphragm A-bands differentially. Without dextran, E_app_ significantly increased to 7.95 ± 0.33 kPa (mean ± SEM) in stretched psoas but only to 4.46 ± 0.53 kPa in stretched diaphragm (Fig. 4e). With dextran present in the relaxing buffer, E_app_ increased to 17.51 ± 1.27 kPa in stretched psoas and significantly less, to 11.35 ± 1.11 kPa, in stretched diaphragm. Interestingly, the lateral stiffness of the Z-disk region, measured for comparison, increased with osmotic lattice compression and with sarcomere strain, but was indifferent to myofibril type (Fig. S1b). Possibly, the known differences in Z-disk structure (and strength?) between fast (psoas) and slow (diaphragm) muscle^34^ offset any effect of titin stiffness on Z-disk lateral stiffness. We conclude that the effects of osmotic compression and titin stiffness increase on lateral stiffness are cumulative (although not necessarily in terms of the arithmetical sum). A certain proportion of the lateral stiffness increase at the A-band should simply be mediated by the reduction in lattice spacing, which probably increases the electrostatic repulsion between the myofilaments. However, the stiffness of titin *per se* is important too, since it affects A-band lateral stiffness (Fig. 4e), but is not a critical factor in the strain-induced compression of the inter-filament lattice (Fig. 2c). To explain this scenario, we consider a direct effect of titin stiffness on proteins within the actin-myosin overlap zone.

Several lines of evidence suggest that various A-band proteins could “sense” titin stiffness. Among these proteins are the myosin heads, which bind to A-band titin modules^35^ and show altered ordering with increasing SL in relaxed muscle^36^, although a positioning closer to actin could not be confirmed in a new study^37^. Furthermore, the stiffening of titin strains the myosin filament by a small degree^11^ and this modification could translate into altered A-band lateral stiffness. Other candidate proteins are the troponin subunits and tropomyosin, which both regulate the activation of the thin filament and are important for LDA^2^. Because the titin spring binds to sarcomeric actin^38^,^39^ and tropomyosin^40^, stretch of titin could in theory affect the structural arrangement of these thin filament proteins, perhaps with long-range effects on their conformation in the A-band region. Notably, a special type of LDA, the stretch activation of insect indirect flight muscle, is mediated by a troponin isoform that forms bridges with myosin subunits^41^,^42^. If similar connections were present in mammalian sarcomeres, and if they were somehow engaged by the stretching of the titin filaments, one could envision such troponin bridges to mediate the effect of titin stiffness increase on lateral A-band stiffness. An additional candidate is myosin-binding protein-C (MyBP-C), which binds to myosin, titin, and actin^43^. Conceivably, increased titin stiffness from sarcomere strain could impact the positioning of MyBP-C between the actin and myosin filaments and thus, affect lateral A-band stiffness.

In summary, our work provides evidence for a direct effect of titin stiffness on A-band lateral stiffness, which appears under sarcomere strain. This effect is largely independent of changes in inter-filament spacing. The titin contribution to A-band lateral stiffness is additive to the contribution from ionic interactions between actin and myosin filaments probed by osmotic compression. Taken together with previous findings, including latest data from time-resolved small-angle X-ray diffraction measurements on cardiac muscle^37^, it appears that the sarcomere can sense titin stiffness via structural re-arrangements or conformational changes of proteins within the actin-myosin overlap zone. These effects may provide, in part, the molecular basis for LDA. Our study thus corroborates other recent evidence in favour of an active involvement of titin in muscle contraction^44^,^45^. As titin is increasingly recognized as an important (cardio)myopathy gene/protein, our findings also have implications for a better understanding of the pathophysiology of heart and muscle diseases.

## Methods

### Myofibril stretch experiments and A-band diameter measurements

Myofibrils were prepared from rabbit psoas and diaphragm muscle as described^15^. Briefly, muscle strips were dissected, tied to glass rods and used up fresh or stored at −20 °C in rigor buffer (75 mM KCl, 10 mM Tris, 2 mM MgCl_2_, 2 mM EGTA, pH 7.1; 40 μg/mL protease inhibitor leupeptin) containing 50% glycerol. Experimental procedures were performed in accordance with the Guide for the Care and Use of Laboratory Animals published by the US National Institutes of Health (8^th^ Edition, 2011). All experimental protocols were approved by the Institutional Animal Care and Use Committee of Ruhr University Bochum. To obtain myofibrils, small muscle pieces were minced, exposed for ~5 h to rigor buffer containing 0.5% Triton X-100 for skinning, and homogenized in rigor buffer. A drop of the myofibril suspension was placed on a cover slip under a Zeiss Axiovert 135 microscope. A single myofibril was selected and glued at the ends to the tip of a fine glass micropipette controlled by a micromanipulator (MHW 103, Narishige). Surplus myofibrils were removed by several washes with rigor buffer or relaxing buffer (7.8 mM ATP, 10 mM phosphocreatine, 20 mM imidazole, 4 mM EGTA, 12 mM Mg-propionate, 97.6 mM K-propionate, pH 7.0, 40 μg/ml leupeptin, 1 mM DTT; ionic strength,180 mM). In a subset of experiments, we added 5% dextran T-500 (w/v) to the relaxing buffer as an osmotic agent. All experiments were performed at room temperature. Using a custom-made setup for isolated myofibril mechanics^46^,^47^, a single myofibril was stretched stepwise in relaxing buffer with or without dextran. At each stretch state the myofibril image was recorded using an 885-EMCCD camera (Andor Technology), the SL was determined, and the A-band diameter of at least five sarcomeres/myofibril was measured (ImageJ software; NIH, Bethesda) by calculating the full-width-at-half-maximum (FWHM) signal on intensity profiles perpendicular to the myofibril axis.

### Passive force measurements on single myofibres

Psoas or diaphragm muscle fibres were dissected from Triton-skinned muscle bundles. Dissection was done in relaxing buffer and care was taken to avoid excessive stretching of the fibres. Single fibres were suspended in a muscle mechanics workstation (Scientific Instruments, Heidelberg) and force-extension measurements were performed in relaxing buffer at room temperature, as described^15^. Fibres were stretched from slack SL (2.0–2.2 μm) in 5 steps of ~0.2 μm/sarcomere (completed in 1 s), while force was recorded (sampling rate, 100 Hz) and SL measured by laser diffraction during a 1-min hold period after each step. Following the last stretch-hold, fibres were released back to slack SL to test for possible shifts of baseline force. Three identical stretch-release protocols were performed on the same fibre (with a 5-min pause in-between) and the mean forces calculated. The force value at the end of each hold period (quasi steady-state force) was used to determine force per cross-sectional area (stress). Fibre cross-sectional area was estimated from the diameter (assuming a circular shape) measured at slack SL under a binocular microscope using a μm-grid. Following a series of measurements in relaxing buffer, 5% dextran T-500 was added to the solution and another series of measurements performed on the same fibre.

### Titin gels

Titin isoforms were separated as described^48^. Briefly, tissue samples from the muscles used for mechanical measurements were solubilized in 50 mM Tris-sodium dodecyl sulfate (SDS) buffer (pH 6.8) containing 8 μg/mL leupeptin (Peptin Institute, Japan) and phosphatase inhibitor cocktail (PIC [P2880], 10 μL/mL; Sigma). Samples were heated for 3 minutes and centrifuged. Psoas, diaphragm, and a mix of both muscles were loaded at low (15 μg) and high (30 μg) concentrations and separated by 1.8% SDS polyacrylamide gel electrophoresis. Gels were run at 5 mA constant current for 16 hours. Titin bands were visualized by Coomassie-staining and digitized using LAS-4000 Image Reader (Fujifilm).

### AFM imaging of myofibrils and topographical analysis of putative myosin filaments

Glass cover slips were extensively washed and incubated in 5 M KOH for 30 minutes. Then the slides were sonicated, washed with ethanol and air dried. Psoas myofibrils in relaxing buffer were placed on a cover slip and height images of well-adhering single myofibrils or doublets were acquired (scan rate, 1 Hz; 256 pixels/scan) using an integrated AFM/inverted optical microscope (MFP-3D-BIO, Asylum Research). Rectangular, gold-coated silicon nitride cantilevers (Biolever BL-RC150VB-C1 (A lever) from Olympus; length, 60 μm; spring constant, 0.03 N/m; resonant frequency, 37 kHz (in air)) with a V-shaped tip (apex diameter, 60 nm; height, 7 μm) were used. Tip geometry resembles a hollow pyramid sliced in half vertically, with a sharpened apex, allowing for exact positioning on the myofibril. To minimize tip/sample interaction, tapping mode was used. Setpoint amplitude and integral gain were carefully adjusted for each experiment, following published protocols^33^. Line scans recorded perpendicular to the myofibril axis (in the A-band region) were searched for clear peaks representing elevated structures on the myofibrillar surface, presumably indicating the myosin filaments. The peak-to-peak distances were determined and collectively used to infer the primary myosin spacing (A_p_) and the inter-filament distance, d_1,0_, using d_1,0_ = A_P_ · √3/2, as described^25^. Furthermore, an algorithm was used that detected the peaks in each line scan, followed them in 50–100 line scans/A-band image, and calculated all nearest peak-to-peak (=inter-filament) distances.

### AFM force mapping and analysis of lateral stiffness

Myofibrils adhering to the cover slip were analyzed for their lateral stiffness in rigor buffer and in relaxing buffer with or without dextran. We studied relaxed myofibrils either at slack SL or following a controlled stretch to ~145% slack, performed under a Zeiss Axiovert 145 inverted microscope. The stretched myofibril was glued at the ends to the cover glass using silicone adhesive (1:1 mixture (v/v) of Dow Corning 3145 RTV and 3140 RTV) and the glass needle tips to which the myofibril was attached were broken by pressing the tips onto the glass surface. The stretched myofibril immersed in relaxing buffer at any time was then transferred together with the cover slip to the AFM. For force mapping, we first measured the resonant frequency (~8 kHz) and the spring constant (~0.03 N/m) of the Biolever BL-RC150VB-C1 (A lever) in relaxing buffer, followed by a short scan in contact mode to ensure an intact and clean cantilever tip. After selecting a region of interest containing a whole sarcomere, 2,500 (50 × 50) individual force curves were recorded over this region to generate a force (stiffness) map. Individual force curves were analyzed as follows^25^. The contact point was identified using the second derivative of the filtered deflection-extension curve and the origin of the curve was set to this point. The indentation *δ* was computed by subtracting the deflection *d* of the cantilever from the piezo movement *z* after contacting the myofibril (*δ* = *z* − *d*), to obtain force-indentation curves. Since the myofibrils have nonlinear transversal elastic moduli, we calculated the pointwise apparent modulus, E_app_, to obtain depth-dependent changes in the lateral stiffness of the sarcomere, using F = 2π × E_app_ × *Ø*(*δ*), where F is the indentation force and *Ø*(*δ*) expresses the geometry of the cantilever tip^33^. For *Ø*(*δ*) we used the equation for a parabolic indenter, *Ø*(*δ*) = 4/3π × √(R_C_*δ*^3^), with R_C_ being the radius of curvature of the tip (~50 nm). E_app_ is a measure of lateral stiffness and can be related to the Young’s modulus E via E = E_app_ × 2(1 − *ν*^2^)], where *ν* is the Poisson ratio. (For biological samples, *ν* is usually set to 0.5, assuming a constant volume.) For the analysis we used the E_app_ values at 60–100 nm indentation, because E_app_ approached a plateau at this indentation depth. E_app_ was determined in the actin-myosin overlap region of a given sarcomere (=A-band region) and also in the Z-disk region. The A-band, M-band, and Z-disk regions were detected by their different lateral stiffness and by their different surface topology measured on AFM height images acquired in tapping mode following the force mapping experiment. Only those sarcomeres were included in the analysis where the Z-disk stiffness could readily be distinguished from the lateral stiffness of the remainder of the sarcomere. In relaxed sarcomeres at slack length, the Z-disk was usually the stiffest sarcomeric region.

### Statistics

Results were depicted as mean ± SEM, unless indicated otherwise. Significance was tested by Bonferroni-adjusted t-test in conjunction with ANOVA. Three levels of significance were used: *p < 0.05; **p < 0.01; and ***p < 0.001.

## Additional Information

**How to cite this article**: Li, Y. *et al.* Titin stiffness modifies the force-generating region of muscle sarcomeres. *Sci. Rep.* **6**, 24492; doi: 10.1038/srep24492 (2016).

## Supplementary Material

Supplementary Information

## References

[b1] de Tombe P. P. *et al.* Myofilament length dependent activation. J. Mol. Cell. Cardiol. 48, 851–858 (2010). 20053351 10.1016/j.yjmcc.2009.12.017 PMC2854194

[b2] Kobirumaki-Shimozawa F. *et al.* Cardiac thin filament regulation and the Frank-Starling mechanism. J. Physiol. Sci. 64, 221–232 (2014). 24788476 10.1007/s12576-014-0314-y PMC4070490

[b3] Kobirumaki-Shimozawa F. *et al.* Nano-imaging of the beating mouse heart *in vivo*: Importance of sarcomere dynamics, as opposed to sarcomere length per se, in the regulation of cardiac function. J. Gen. Physiol. 147, 53–62 (2016). 26712849 10.1085/jgp.201511484 PMC4692490

[b4] Konhilas J. P., Irving T. C. & de Tombe P. P. Length-dependent activation in three striated muscle types of the rat. J. Physiol. 544, 225–236 (2002). 12356894 10.1113/jphysiol.2002.024505 PMC2290573

[b5] Godt R. E. & Maughan D. W. Influence of osmotic compression on calcium activation and tension in skinned muscle fibers of the rabbit. Pflugers Arch. 391, 334–337 (1981). 7312568 10.1007/BF00581519

[b6] McDonald K. S. & Moss R. Osmotic compression of single cardiac myocytes eliminates the reduction in Ca2+ sensitivity of tension at short sarcomere length. Circ. Res. 77, 199–205 (1995). 7788878 10.1161/01.res.77.1.199

[b7] Fuchs F. & Smith S. H. Calcium, cross-bridges, and the Frank-Starling relationship. News Physiol. Sci. 16, 5–10 (2001). 11390938 10.1152/physiologyonline.2001.16.1.5

[b8] Smith D. A. Electrostatic forces or structural scaffolding: what stabilizes the lattice spacing of relaxed skinned muscle fibers? J. Theor. Biol. 355, 53–60 (2014). 24703982 10.1016/j.jtbi.2014.03.037

[b9] Konhilas J. P., Irving T. C. & de Tombe P. P. Myofilament calcium sensitivity in skinned rat cardiac trabeculae: role of inter-filament spacing. Circ. Res. 90, 59–65 (2002). 11786519 10.1161/hh0102.102269

[b10] Cazorla O., Wu Y., Irving T. C. & Granzier H. Titin-based modulation of calcium sensitivity of active tension in mouse skinned cardiac myocytes. Circ. Res. 88, 1028–1035 (2001). 11375272 10.1161/hh1001.090876

[b11] Irving T. *et al.* Thick-filament strain and interfilament spacing in passive muscle: effect of titin-based passive tension. Biophys. J. 100, 1499–1508 (2011). 21402032 10.1016/j.bpj.2011.01.059 PMC3059568

[b12] Methawasin M. *et al.* Experimentally increasing titin compliance in a novel mouse model attenuates the Frank-Starling mechanism but has a beneficial effect on diastole. Circulation 129, 1924–1936 (2014). 24599837 10.1161/CIRCULATIONAHA.113.005610 PMC4032222

[b13] Fukuda N., Sasaki D., Ishiwata S. & Kurihara S. Length dependence of tension generation in rat skinned cardiac muscle: role of titin in the Frank-Starling mechanism of the heart. Circulation. 104, 1639–1645 (2001). 11581142 10.1161/hc3901.095898

[b14] Fukuda N. *et al.* Sarcomere length-dependent Ca2+ activation in skinned rabbit psoas muscle fibers: coordinated regulation of thin filament cooperative activation and passive force. J. Physiol. Sci. 61, 515–523 (2011). 21901640 10.1007/s12576-011-0173-8 PMC3204045

[b15] Prado L. G. *et al.* Isoform diversity of giant proteins in relation to passive and active contractile properties of rabbit skeletal muscles. J. Gen. Physiol. 126, 461–480 (2005). 16230467 10.1085/jgp.200509364 PMC2266601

[b16] Kaasik A. *et al.* Mitochondria as a source of mechanical signals in cardiomyocytes. Cardiovasc. Res. 87, 83–91 (2010). 20124402 10.1093/cvr/cvq039

[b17] Farman G. P., Walker J. S., de Tombe P. P. & Irving T. C. Impact of osmotic compression on sarcomere structure and myofilament calcium sensitivity of isolated rat myocardium. Am. J. Physiol. Heart Circ. Physiol. 291, H1847–H1855 (2006). 16751283 10.1152/ajpheart.01237.2005

[b18] Allen J. D. & Moss R. L. Factors influencing the ascending limb of the sarcomere length-tension relationship in rabbit skinned muscle fibres. J. Physiol. 390, 119–136 (1987). 2450989 10.1113/jphysiol.1987.sp016689 PMC1192169

[b19] Kawai M., Wray J. S. & Zhao Y. The effect of lattice spacing change on cross-bridge kinetics in chemically skinned rabbit psoas muscle fibers. I. Proportionality between the lattice spacing and the fiber width. Biophys. J. 64, 187–196 (1993). 7679296 10.1016/S0006-3495(93)81356-0 PMC1262316

[b20] Wang Y. P. & Fuchs F. Length-dependent effects of osmotic compression on skinned rabbit psoas muscle fibers. J. Muscle Res. Cell Motil. 21, 313–319 (2000). 11032342 10.1023/a:1005679215704

[b21] Llewellyn M. E., Barretto R. P., Delp S. L. & Schnitzer M. J. Minimally invasive high-speed imaging of sarcomere contractile dynamics in mice and humans. Nature. 454, 784–788 (2008). 18600262 10.1038/nature07104 PMC2826360

[b22] Martyn D. A. & Gordon A. M. Length and myofilament spacing-dependent changes in calcium sensitivity of skeletal fibres: effects of pH and ionic strength. J. Muscle Res. Cell Motil. 9, 428–445 (1988). 3215997 10.1007/BF01774069

[b23] Berman M. R. & Maughan D. W. Axial elastic modulus as a function of relative fiber width in relaxed skinned skeletal muscle fibers. Pflugers Arch. 393, 99–103 (1982). 6178081 10.1007/BF00582400

[b24] Shimamoto Y., Kono F., Suzuki M. & Ishiwata S. Nonlinear force-length relationship in the ADP-induced contraction of skeletal myofibrils. Biophys. J. 93, 4330–4341 (2007). 17890380 10.1529/biophysj.107.110650 PMC2098727

[b25] Nyland L. R. & Maughan D. W. Morphology and transverse stiffness of Drosophila myofibrils measured by atomic force microscopy. Biophys. J. 78, 1490–1497 (2000). 10692334 10.1016/S0006-3495(00)76702-6 PMC1300747

[b26] Millman B. M. The filament lattice of striated muscle. Physiol. Rev. 78, 359–391 (1998). 9562033 10.1152/physrev.1998.78.2.359

[b27] Brenner B. & Yu L. C. Characterization of radial force and radial stiffness in Ca(2+)-activated skinned fibres of the rabbit psoas muscle. J. Physiol. 441, 703–718 (1991). 1816390 10.1113/jphysiol.1991.sp018774 PMC1180221

[b28] Xu S., Martyn D., Zaman J. & Yu L. C. X-ray diffraction studies of the thick filament in permeabilized myocardium from rabbit. Biophys. J. 91, 3768–3775 (2006). 16950853 10.1529/biophysj.106.088971 PMC1630466

[b29] Radmacher M., Cleveland J. P., Fritz M., Hansma H. G. & Hansma P. K. Mapping interaction forces with the atomic force microscope. Biophys. J. 66, 2159–2165 (1994). 8075349 10.1016/S0006-3495(94)81011-2 PMC1275941

[b30] Yoshikawa Y., Yasuike T., Yagi A. & Yamada T. Transverse elasticity of myofibrils of rabbit skeletal muscle studied by atomic force microscopy. Biochem. Biophys. Res. Commun. 256, 13–19 (1999). 10066415 10.1006/bbrc.1999.0279

[b31] Akiyama N., Ohnuki Y., Kunioka Y., Saeki Y. & Yamada T. Transverse stiffness of myofibrils of skeletal and cardiac muscles studied by atomic force microscopy. J. Physiol. Sci. 56, 145–151 (2006). 16839448 10.2170/physiolsci.RP003205

[b32] Miyashiro D., Wakayama J., Akiyama N., Kunioka Y. & Yamada T. Radial stability of the actomyosin filament lattice in isolated skeletal myofibrils studied using atomic force microscopy. J. Physiol. Sci. 63, 299–310 (2013). 23690090 10.1007/s12576-013-0268-5 PMC10717890

[b33] Azeloglu E. U. & Costa K. D. Cross-bridge cycling gives rise to spatiotemporal heterogeneity of dynamic subcellular mechanics in cardiac myocytes probed with atomic force microscopy. Am. J. Physiol. Heart Circ. Physiol. 298, H853–H860 (2010). 20023124 10.1152/ajpheart.00427.2009

[b34] Rowe R. W. The ultrastructure of Z-disks from white, intermediate, and red fibers of mammalian striated muscles. J. Cell Biol. 57, 261–277 (1973). 4696547 10.1083/jcb.57.2.261 PMC2108976

[b35] Muhle-Goll C. *et al.* Structural and functional studies of titin’s fn3 modules reveal conserved surface patterns and binding to myosin S1–a possible role in the Frank-Starling mechanism of the heart. J. Mol. Biol. 313, 431–447 (2001). 11800567 10.1006/jmbi.2001.5017

[b36] Farman G. P. *et al.* Myosin head orientation: a structural determinant for the Frank-Starling relationship. Am. J. Physiol. Heart Circ. Physiol. 300, H2155–H2160 (2011). 21460195 10.1152/ajpheart.01221.2010 PMC3119094

[b37] Ait-Mou Y. *et al.* Titin strain contributes to the Frank-Starling law of the heart by structural rearrangements of both thin- and thick-filament proteins. Proc. Natl. Acad. Sci. USA. 113, 2306–2311 (2016). 26858417 10.1073/pnas.1516732113 PMC4776536

[b38] Kulke M. *et al.* Interaction between PEVK-titin and actin filaments: origin of a viscous force component in cardiac myofibrils. Circ Res. 89, 874–881 (2001). 11701614 10.1161/hh2201.099453

[b39] Linke W. A. *et al.* PEVK domain of titin: an entropic spring with actin-binding properties. J. Struct. Biol. 137, 194–205 (2002). 12064946 10.1006/jsbi.2002.4468

[b40] Raynaud F., Astier C. & Benyamin Y. Evidence for a direct but sequential binding of titin to tropomyosin and actin filaments. Biochim. Biophys. Acta 1700, 171–178 (2004). 15262226 10.1016/j.bbapap.2004.05.001

[b41] Agianian B. *et al.* A troponin switch that regulates muscle contraction by stretch instead of calcium. EMBO J. 23, 772–779 (2004). 14765112 10.1038/sj.emboj.7600097 PMC381005

[b42] Perz-Edwards R. J. *et al.* X-ray diffraction evidence for myosin-troponin connections and tropomyosin movement during stretch activation of insect flight muscle. Proc. Natl. Acad. Sci. USA 108, 120–125 (2011). 21148419 10.1073/pnas.1014599107 PMC3017141

[b43] Sadayappan S. & de Tombe P. P. Cardiac myosin binding protein-C as a central target of cardiac sarcomere signaling: a special mini review series. Pflugers Arch. 466, 195–200 (2014). 24196566 10.1007/s00424-013-1396-8 PMC3946865

[b44] Rivas-Pardo J. A. *et al.* Work done by titin protein folding assists muscle contraction. Cell Rep. 14, 1339–1347 (2016). 26854230 10.1016/j.celrep.2016.01.025 PMC4865255

[b45] Hinson J. T. *et al.* Titin mutations in iPS cells define sarcomere insufficiency as a cause of dilated cardiomyopathy. Science. 349, 982–986 (2015). 26315439 10.1126/science.aaa5458 PMC4618316

[b46] Linke W. A., Ivemeyer M., Mundel P., Stockmeier M. R. & Kolmerer B. Nature of PEVK-titin elasticity in skeletal muscle. Proc. Natl. Acad. Sci. USA 95, 8052–8057 (1998). 9653138 10.1073/pnas.95.14.8052 PMC20927

[b47] Opitz C. A. *et al.* Damped elastic recoil of the titin spring in myofibrils of human myocardium. Proc. Natl. Acad. Sci. USA 100, 12688–12693 (2003). 14563922 10.1073/pnas.2133733100 PMC240679

[b48] Hamdani N. *et al.* Crucial role for Ca2+/calmodulin-dependent protein kinase-II in regulating diastolic stress of normal and failing hearts via titin phosphorylation. Circ. Res. 112, 664–674 (2013). 23283722 10.1161/CIRCRESAHA.111.300105

