# Supplementary material for: Titin stiffness modifies the force-generating region of muscle sarcomeres
Source: Sci Rep. 2016 Apr 15;6:24492. doi: 10.1038/srep24492 (PMC4832248; doi:10.1038/srep24492)
Supplement: Supplementary Information [file srep24492-s1.pdf]

Supplemental Information

Titin stiffness modifies the force-generating region of muscle sarcomeres

Yong Li[#], Patrick Lang[#] & Wolfgang A. Linke*

Department of Cardiovascular Physiology, Ruhr University Bochum, Germany

*Corresponding author:

Wolfgang A. Linke, PhD

Dept. of Cardiovascular Physiology

Ruhr University Bochum, MA 03/56

D-44780 Bochum, Germany

Email: wolfgang.linke@rub.de

[#]Co-first authors

Figure S1, related to Figure 4

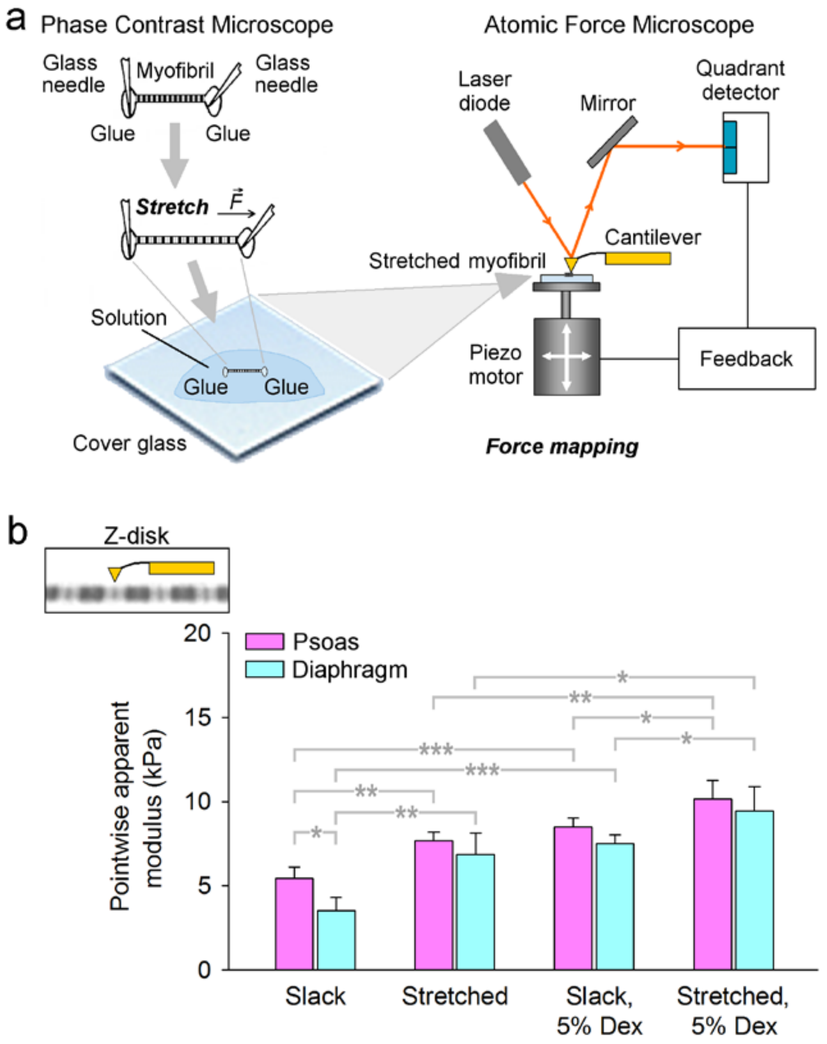

Figure S1. AFM-based force mapping of sarcomeres to measure lateral stiffness. (a) Workflow for force mapping experiments on stretched myofibrils. (b) Pointwise apparent modulus (at 60-100 nm indentation depth) to quantify the lateral stiffness of the Z-disk region of psoas and diaphragm sarcomeres in relaxing buffer at slack SL; ~145% stretch; slack SL, 5% dextran; and ~145% stretch, 5% dextran (n=5 sarcomeres analyzed per group). Data are mean±SEM; *$p<0.05$; **$p<0.01$; ***$p<0.001$, by ANOVA/Bonferroni-adjusted t-test.
